# Attachment styles among Tunisian women: cross-sectional study

**DOI:** 10.1192/j.eurpsy.2025.598

**Published:** 2025-08-26

**Authors:** N. H. Benhamed, A. Hakiri, Y. Yaich, B. Amamou

**Affiliations:** 1 Psychiatry department, EPS Fatouma Bourguiba, Monastir; 2 Psychiary B. Razi Hospital, Manouba, Tunisia

## Abstract

**Introduction:**

Attachment styles are essential for understanding how individuals form and maintain emotional connections and relationships, significantly influencing emotional well-being and interpersonal dynamics. In Tunisia, where cultural expectations around gender and relationships play a prominent role, exploring attachment styles provides insight into how women navigate intimacy, trust, and emotional security.

**Objectives:**

This study aims to assess the prevalence of attachment styles among Tunisian women and investigate their associations with various sociodemographic and psychological factors

**Methods:**

a cross-sectional study was conducted online using a Google Forms questionnaire between July and August 2024.The inclusion criteria were sexually active women aged 18 years or older who provided informed consent to participate. Participants completed a self-administered questionnaire that included sociodemographic information, personal medical history, lifestyle habits, and psychometric assessments. The relationship Questionaire (RQ) was used to evaluate attachement style and self-esteem was assessed using the Rosenberg Self-Esteem Scale (RSE).

**Results:**

A total of 180 women participated in the study, with an average age of 32.79 years, ranging from 21 to 60 years. The majority, 97.78%, resided in urban areas, while 94.44% held a university degree, and 80% were employed. Medical history revealed that 21.11% reported organic issues, and 27.22% had a psychiatric history. Lifestyle habits indicated that 18.9% of women smoked, 21.1% consumed alcohol, and only 1.1% used psychoactive substances.

Evaluation of attachment styles showed that 57.78% of women exhibited a secure attachment style (n=104), 26.67% had an avoidant style (n=48), 8.89% displayed a disorganized style (n=16), and 6.67% had an anxious style (n=12). Attachment styles were significantly associated with age (p=0.003), employment status (p=0.004), marital status (p=0.01), organic health issues (p=0.02), history of suicide attempts (p<0.001), self-esteem (p<0.001), and the number of sexual partners (p=0.01).

**Conclusions:**

The findings highlight that attachment styles are significantly linked to several sociodemographic and psychological factors among Tunisian women. Understanding these associations is crucial for developing targeted interventions that promote secure attachment patterns, improve emotional well-being, and enhance relationship quality. By addressing these dynamics, we can foster psychological resilience and support healthier relational patterns. Further research could extend these insights to broader sociocultural contexts, offering more tailored approaches to improving women’s mental health and relational satisfaction.

**Disclosure of Interest:**

None Declared

